# Oleuropein Degradation Kinetics in Olive Leaf and Its Aqueous Extracts

**DOI:** 10.3390/antiox10121963

**Published:** 2021-12-08

**Authors:** María Esther Martínez-Navarro, Cristina Cebrián-Tarancón, José Oliva, María Rosario Salinas, Gonzalo L. Alonso

**Affiliations:** 1 Cátedra de Química Agrícola, E.T.S.I. Agrónomos y Montes, Departamento de Ciencia y Tecnología Agroforestal y Genética, Universidad de Castilla-La Mancha, Avda. de España s/n, 02071 Albacete, Spain; Mesther.Martinez@uclm.es (M.E.M.-N.); cristina.ctarancon@uclm.es (C.C.-T.); Rosario.Salinas@uclm.es (M.R.S.); 2 Departamento de Química Agrícola, Geología y Edafología, Facultad de Química, Universidad de Murcia, Campus de Espinardo s/n, 30100 Murcia, Spain; josoliva@um.es

**Keywords:** by-product, kinetics, liquid extract, oleuropein, olive leaves, storage

## Abstract

Although olives leaves are currently considered a waste material from oil mills, they have great potential to be transformed into by-products due to their high oleuropein content. Oleuropein is a glycoside precursor of hydroxytyrosol, which is the phenolic compound with the highest antioxidant capacity in nature and which is associated with multiple health benefits. For this reason, the demand for oleuropein is growing in the pharmaceutical, cosmetic and food sectors. The objective of this study is to determine the stability of oleuropein in olive leaves from oil mills in solid and aqueous forms under different conditions of temperature, relative humidity and lighting. The results indicate that the degradation of oleuropein conforms well to first-order kinetics. The rate constants at the temperatures tested in the aqueous extracts indicate activation energies from RT_l_ to 80 °C and from 7 °C to 14 °C, as the degradation reactions were different in these ranges. Furthermore, olive leaf powder stored at any temperature with an RH ≥ 57% showed greater stability after six months, which is an encouraging result for the storage and transformation of this waste in oil mills.

## 1. Introduction

Olive leaves (*Olea europaea* L.) are still considered a waste product produced by oil mills, especially given the pruning and maintenance labours in grove olive. During the pruning season, 1.5–3 annual tons of leaves per ha can be collected, while the weight of leaves in an oil mill ranges from approximately 0.075 to 0.15 annual tons per ha [1,2]. Traditionally, these waste products are burned or used for animal feed. However, the olive leaves are rich in polyphenols, bioactive compounds with beneficial health effects, which should be used as secondary raw materials for pharmaceutical, cosmetic and food sectors [3,4]. These health benefits are due to the composition in bioactive compounds, mainly oleuropein. The latter is the most abundant of these compounds and, to a lesser extent, hydroxycinnamic-acid derivatives such as verbascoside; simple phenolic alcohols, such as hydroxytyrosol and tyrosol; and flavonoids, such as apigenin 7-*O*-glucoside and diosmetin-7-*O*-glucoside. Oleuropein is the glycoside formed between a molecule of elenolic acid linked to hydroxytyrosol by an ester bond and to a molecule of glucose by a glycosidic bond. Therefore, oleuropein has been identified as the most suitable precursor to hydroxytyrosol, which has a wide range of biotic and pharmacological uses [5]. The antioxidant properties of products and by-products from an olive tree have been categorically proven, demonstrating that oleuropein and hydroxytyrosol are especially potent scavengers of superoxide anion and other reactive species [6]. Hydroxytyrosol is considered the most powerful antioxidant compound after gallic acid and one of the most powerful antioxidant compounds alongside phenolic and oleuropein compounds [7]. Due to its high antioxidant capacity, it is a potential therapeutic, antithrombotic, cardioprotective, antitumor microbicide and anti-inflammatory agent [8,9,10,11,12]. Furthermore, a health claim on olive oil polyphenols from the European Food Safety Authority states that they contribute to the protection of blood lipids from oxidative stress [13]. For these reasons, such compounds have attracted great interest from the pharmaceutical, cosmetic and food sectors.

Oleuropein, hydroxytyrosol and other olive leaf bioactive compounds are soluble in water, which gives oil mills great potential to obtain aqueous extracts rich in bioactive compounds and possibly transform this waste into a by-product. There is also the possibility of transforming the leaves in a solid form into by-products

However, no studies have focused on how the bioactive compounds in the olive leaf could evolve if they were transformed into a by-product in the olive mill and stored there. Thus, the aim of this study is to determine the stability of the oleuropein content in olive leaves derived from oil mills in both solid and aqueous forms under different conditions of temperature, relative humidity and lighting.

## 2. Materials and Methods

### 2.1. Plant Material 

Olive leaves (*Olea europaea* L.) were obtained from the processing line of an oil mill located in the Castilla-La Mancha region of southwest Spain (altitude of 910 m, 38°41′8″ N latitude and 2°29′26″ W longitude) in November 2020. The leaves were air-dried in an uncontrolled room temperature in the dark for seven days. The samples were then stored at room temperature until use.

The dried leaves were ground in a knife mill (ARES FML-2000; Filtra Vibración, Barcelona, Spain) for 30 s and passed through a sieve (500 mesh) until at least 95% of the total weight had passed through, yielding olive leaf powder (solid form).

### 2.2. Storage Conditions 

The solid storage conditions were based on those used by Moratalla-López et al. (2019) [14]: room temperature (RT_s_; 22.8 ± 1.9 °C) and 40 °C; three relative humidities (RH; 23%, 57% and 75%); and two lighting conditions (natural light and darkness). The laboratory room was set to 22 °C, and the laboratory oven was set to 40 °C. Each laboratory oven held three hermetically sealed boxes containing different saturated solutions (potassium acetate, sodium bromide and sodium chloride) to produce 23%, 57% and 75% RH, respectively. Twelve glass petri dishes measuring 6 cm in diameter and 0.2 cm thick were used with 15 g of olive leaf powder. The hermetically sealed boxes had dimensions of 25 × 15 × 7.5 cm (length × width × height). Four glass petri dishes of olive leaf powder were placed in each box and were separated from the corresponding saturated solution by a grid. In addition, two petri dishes were stored at RT_s_ and ambient humidity.

Liquid storage conditions were 80, 60, 40, RT_l_ (24 ± 1.4 °C), 14 and 7 °C. Laboratory ovens were set at 80, 60 and 40 °C, while the refrigerators were set at 14 and 7 °C. Four bottles with 30 mL of aqueous olive leaf extract were stored at each temperature for high-performance liquid chromatograph with a diode-array detector (HPLC-DAD) and pH analyses. 

### 2.3. Preparation of Oleuropein-Rich Aqueous Extracts from Olive Leaves

Aqueous extracts of olive leaves were prepared according to Martínez-Navarro et al. (2021) [4] with 25 mL of distilled water and 50 mg of olive leaf powder. The mixture was extracted in a domestic microwave oven (MS-2819W; Saivod, Madrid, Spain) at 800 watts for 30 s. For the liquid kinetics, 1 L of aqueous extract was prepared, which was stored under the conditions described in Section 2.2.

### 2.4. Compound Analysis

Oleuropein from aqueous extracts was analysed according to the methodology described by Martínez-Navarro et al. (2021) [4]. These extracts were first injected into an Agilent 1200 HPLC (Palo Alto, CA, USA) equipped with a DAD (Agilent G1315D). The latter was coupled to an Agilent ChemStation (Version B.03.01) data-processing station. Separation was performed at 30 °C on a reverse-phase C18 column (Brisa LC2; 250 mm × 4.6 mm, 5 μm particle) purchased from Teknokroma (Barcelona, Spain).

In addition, the compounds generated in the kinetics process were identified using an Agilent 1290 Series II HPLC (Agilent Technologies Deutschland GmbH, Waldbronn, Germany) coupled to an Agilent 6550 Q-TOF (Agilent Technologies Deutschland GmbH, Waldbronn, Germany) with a Jet Stream dual-electrospray ionisation source. Mass spectrometry (MS) data acquisition was performed in negative scan mode. Nitrogen was used for both the drying gas and the sheath gas in the source. The capillary voltage was set to 4000 V. The nozzle voltage was set to 500 V, and the fragmentor voltage was set to 350 V. The drying gas flow was set to 16 L/min at 150 °C. The sheath gas flow was set to 12 L/min at 300 °C, and the nebuliser was set to 30 psig. The scan range was set to *m*/*z* 50–1100 for the MS and MS/MS modes. To minimise any changes in the compounds’ retention time and to avoid the formation of adducts, we used the same column, solvents, flow rates and elution gradients as were used in the HPLC-DAD analysis. The possible compounds generated in the kinetic process sought were oleuropein (539.177 *m*/*z* [M − H]^−^), hydroxytyrosol hexoside (315.1085 *m*/*z* [M − H]^−^), hydroxytyrosol (153.0557 *m*/*z* [M − H]^−^), hydroxyoleuropein (557.1719 *m*/*z* [M − H]^−^), luteolin (447.0933 *m*/*z* [M − H]^−^), oleoside 11-methyl ester (403.1246 *m*/*z* [M − H]^−^), verbascoside (623.1981 *m*/*z* [M − H]^−^), apigenin-7-glucoside (431.0984 *m*/*z* [M − H]^−^), diosmetin-7-glucoside (461.1089 *m*/*z* [M − H]^−^) and tyrosol (138.164 *m*/*z* [M − H]^−^).

### 2.5. Kinetics Studies

A trial-and-error method was used to find the reaction order. If the assumed order is correct, the appropriate plot of the concentration–time data (zero-order (concentration against time), first-order (ln concentration against time), and second-order (1/concentration against time)) should be linear. The result showing the best correlation coefficient (R^2^) was selected. To obtain the kinetic parameters, from each reaction were obtained reaction order, rate constants (*k*) and half-life periods (t_1/2_) [15]. To perform these calculations, Excel (Office, Microsoft; 2019) was used. 

### 2.6. Statical Analysis

A one-way analysis of variance (ANOVA) was performed on each determination. Mean values were compared via the Tukey test with a 95% confidence interval to determine significant differences using SPSS 23 for Windows (SPSS INC., Chicago, IL, USA). All analyses were performed in triplicate and expressed as milligrams compound per gram of olive leaf for the solid form and milligrams compound per litre aqueous extract for the liquid form.

## 3. Results

### 3.1. Olive Leaf Powder 

**Figure 1:**
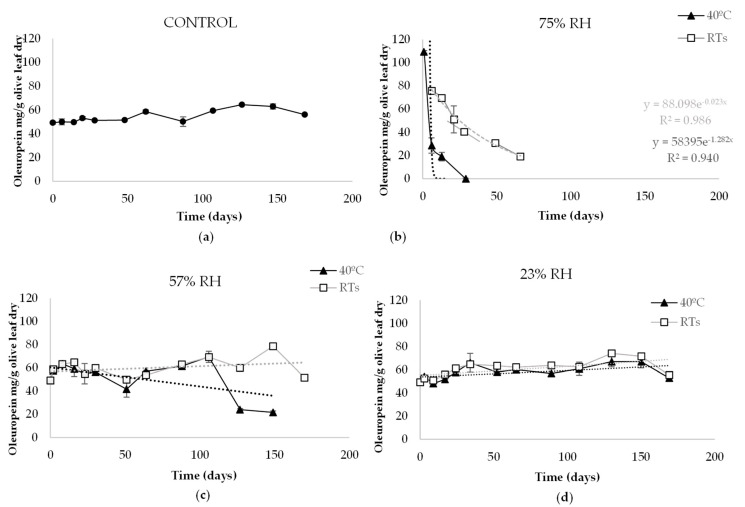
Mean values of oleuropein content (mg/g olive leaf) in olive leaf powder stored at different temperatures (room temperature (RT_s_) and 40 °C) and relative humidities (%RH) for 170 days: (**a**) Control; (**b**) 75% RH; (**c**) 57% RH; (**d**) 23% RH.

The evolution of the oleuropein content in olive leaf powder over time is shown in Figure 1.

At the beginning of the experiment, the oleuropein content was analysed weekly. After a time, the analyses were carried out every three weeks. The initial oleuropein concentration in olive leaf powder was 49.33 mg/g (T0), which evolved in different ways in the studied conditions. In the control olive leaf powder, stored at RT_s_ and environmental humidity (50%), it was observed that all measured concentrations were higher than T0. The maximum oleuropein content, 65.95 mg/g, was reached in the control on Day 147. Regarding 57% RH, an increase in the oleuropein content was also observed after T0 both at RT_s_ and 40 °C. However, on Day 127, the content at 40 °C decreased to 24.07 mg/g, while for the same day at RT_s_, the content was 60.03 mg/g. The maximum content was 78.64 mg/g on Day 149 at 57% RH and RT_s_. Oleuropein content under conditions of 23% RH was the most constant of all those studied along with the control. An increase was also observed after T0, obtaining the highest content of 74.15 mg/g on Day 130 at RT_s_. 

Undoubtedly, the 75% RH storage conditions most affected the oleuropein content (Figure 1b). In fewer than 24 h, the T0 content at 40 °C increased to 109.27 mg/g, which rapidly decreased after six days to 28.58 mg/g. On Day 29, the oleuropein was undetectable. The sample stored at RT_s_ showed a more attenuated tendency. It increased from T0 to 75.62 mg/g, while it decreased over 66 days to 19.11 mg/g. On the following days, no oleuropein content was detected.

The behaviour of oleuropein contained in olive leaf at the different illuminations studied was similar in all storage conditions (data not shown).

### 3.2. Aqueous Olive Leaf Extract

**Figure 2:**
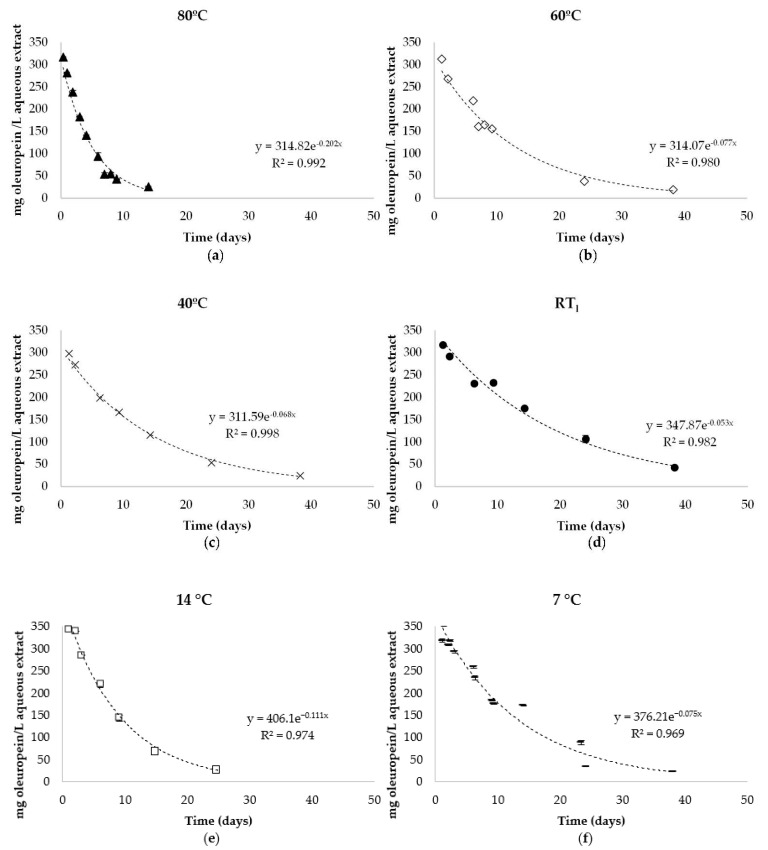
Mean values of oleuropein content (mg/L aqueous extract) in aqueous extract from olive leaf stored at different temperatures for 60 days: (**a**) 80 °C; (**b**) 60 °C; (**c**) 40 °C; (**d**) RT_l_ (24 ± 1.4 °C); (**e**) 14 °C; (**f**) 7 °C.

The starting oleuropein concentration in the aqueous extract was 357.91 mg/L (45.33 mg/g dry olive leaf). Temperature influenced the evolution of the oleuropein; the points obtained fit well to an exponential equation under all storage conditions with an R^2^ between 0.969–0.998 (Figure 2).

**Figure 3:**
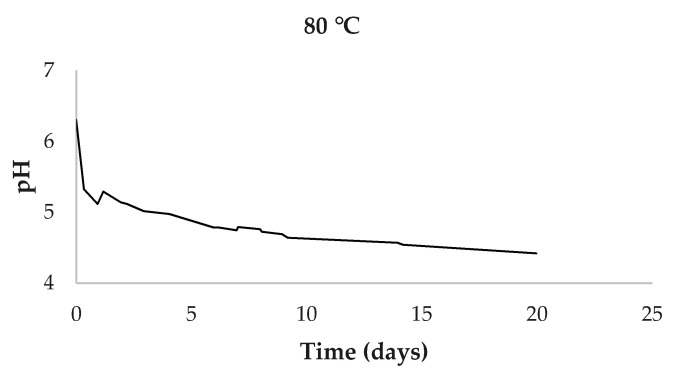
Evolution of mean pH values of aqueous extract at 80 °C.

The pH of the aqueous extracts at the beginning of the experiment was 6.30. This value remained constant at warm and cold storage temperatures. However, at 80 °C, a decrease in pH was observed down to 4.42 (Figure 3). For the other temperatures, the average pH was 5.83 ± 0.29.

### 3.3. Evolution of Compounds 

It was observed that the chemical nature of the compounds in the aqueous extract evolved over time, especially when the conditions were the most adverse. On the other hand, the olive leaf powder in solid form was more stable than the aqueous extracts, except for those stored at 75% RH.

**Figure 4:**
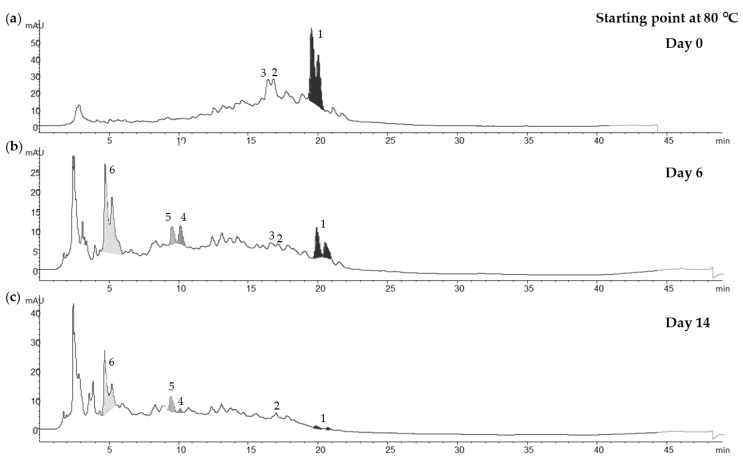
Chromatograms by HPLC-DAD at 280 nm of aqueous olive leaf extracts stored at 80 °C: (**a**) Day 0; (**b**) Day 6; (**c**) Day 14, where 1: oleuropein; 2: verbascoside; 3: hydroxyoleuropein; 4: hydroxytyrosol; 5: hydroxytyrosol hexoside; 6: glycosylated oleuropein derivative (545.1586 *m*/*z* [M − H]^−^_experimental_).

Figure 4 shows the evolution of the liquid extract compounds at 80 °C. The oleuropein was split into two peaks due to the different polarity of the conformational forms of the structure, which was observed by Serrano-Díaz et al. (2014) [16] and others [17] in glycosylated phenolic compounds from plant material. At the beginning (Figure 4a), oleuropein and its conformational isomer (539.1766 *m*/*z* [M − H]^−^_experimental (exp.)_) were predominant, followed by hydroxyoleuropein (555.1735 *m*/*z* [M − H]^−^) and verbascoside (623.2008 *m*/*z* [M − H]^−^_exp._) After six days (Figure 4b), a glycosylated oleuropein derivative appeared, of which the *m*/*z* [M − H]^−^_exp__._ was 545.1586. Hydroxytyrosol hexoside (315.1097 [M − H]^−^_exp._) and hydroxytyrosol (153.0574 [M − H]^−^_exp._) increased, while oleuropein, verbascoside and hydroxyoleuropein decreased. After 14 days (Figure 4c), the oleuropein content was degraded almost completely, while the content of the glycosylated oleuropein derivative compound and the hydroxytyrosol hexoside remained stable. However, hydroxytyrosol began to decline.

On the other hand, the evolution of compounds from olive leaf powder stored at 75% RH, regardless of temperature, showed instability. Oleuropein, hydroxyoleuropein and verbascoside decreased rapidly, whereas hydroxytyrosol hexoside and hydroxytyrosol were below the limits of detection and did not show an increase over time, as happened in the aqueous extracts. As for the glycosylated oleuropein derivative (545.1586 *m*/*z* [M − H]^−^_exp._) found in the aqueous extracts, it was not found in the olive leaf powder (data not shown).

## 4. Discussion

The results obtained from the olive leaf powder showed that the RH influenced (*p* < 0.05) the oleuropein content, decreasing it at a higher RH and keeping it stable at a low RH. This second finding is corroborated by the study carried out by Bilgin et al. [18], who observed a decreasing trend of olive leaf polyphenols in humid air. Temperature was a very influential factor in the oleuropein content (*p* < 0.01) of olive leaf powder. High temperatures can also promote the degradation of some phenolic compounds [19], as was seen at 40 °C and in combination with RH ≥ 57%. Regarding illumination, some authors have related exposure to sunlight with an increase in phenolic compounds in the olive leaf [20]. In contrast, this study showed no significant differences (*p* > 0.05) between storage in darkness and storage in natural light. 

The most unfavourable RH was 75%, both at RT_s_ and 40 °C. The oleuropein content behaved by adjusting to a first-order kinetic model ($y_{RT}=88.098e^{-0.023x}$, R^2^ = 0.986; $y_{40{}^{\circ}C}=58,395e^{-1.282x}$, R^2^ = 0.9399). However, the studied temperatures at 75% RH showed differences in rate constants (k) and half-life periods (t_1/2_). See Table 1. 

At RT_s_, oleuropein content decreased to the level of 19.11 mg/g in 66 days, while at 40 °C, oleuropein was not detected on Day 29 (Figure 1). In other words, the k value was different (k_RT_ = 0.023 and k_40 °C_ = 1.282), which means that, at 75% RH and RT_s_, the t_1/2_ was around 30 days, whereas it was approximately 13 h at 40 °C. During the degradation of the oleuropein content under these conditions, hydroxyoleuropein and verbascoside also decreased rapidly, while the generation of any compound was not observed over time.

By contrast, in aqueous extracts, oleuropein degradation generated other compounds, such as hydroxytyrosol, in the first week of storage. This finding is corroborated by a study carried out by Feng et al. (2021) [21], who observed that, in an olive leaf methanol-water extract, hydroxytyrosol increased in the first week of storage at 25 °C and then decreased after two weeks. This behaviour was observed in the same way, as it was accentuated with the high-temperatures studies. However, at the beginning of the experiment, hydroxytyrosol was found only as a glycosylate compound: hydroxytyrosol hexoside. In addition, there was also an increase in an unknown compound with an *m*/*z* [M − H]^−^ of 545.1586, and this could be directly correlated with the degradation of oleuropein, which occurred simultaneously. Ahmad-Qasem et al. (2016) [22] studied the storage stability of olive leaf extract (liquid or solid) using ethanol–water (80:20 *v*/*v*). They found that neither the extract form nor the storage temperature affected the phenolic content. However, in this study, storage form, humidity and temperature were key to the stability of the oleuropein content in the olive leaf, although other factors such as enzymatic activities are also involved. These latter factors might be responsible for this decrease in oleuropein and increase in other compounds [19]. De Leonardis et al. (2015) [23] found that at least two types of enzymes, β-glucosidase and polyphenoloxidase (PPO), were involved in the degradation of endogenous oleuropein in olive leaves. They observed in aqueous extracts of olive leaves at 60 °C for 24 h that the oleuropein content disappeared completely and increased, mainly, the aldehydic form of oleuropein aglycon (3,4-DHPEA-EA) due to β-glucosidase activity. In contrast, this compound was not observed in our study; instead, a glycosylated oleuropein derivative was formed. They also observed that esterases degraded oleuropein to hydroxytyrosol; however, in their study, the latter remained stable, while the one studied in this work decreased, which could be due to PPO activity. On the other hand, the hydroxytyrosol hexoside in this study remained stable, which could be because these two enzymes cannot act on it. 

In addition, Briante et al. (2001) [24] studied by different methods the antioxidant capacity of oleuropein and hydroxytyrosol, the latter of which showed slightly superior results in all methods. For example, with the DMPD (N,N-dimethyl-p-phenylenediamine dihydrochloride) method, they obtained 0.60 and 0.79 Trolox equivalents for oleuropein and hydroxytyrosol, respectively. The increase in compounds such as hydroxytyrosol and hydroxytyrosol hexoside in the first week points to a higher antioxidant capacity of the liquid extract. On the other hand, although the hydroxytyrosol hexoside remained stable, the degradation of the hydroxytyrosol after 14 days suggests a decrease in antioxidant capacity. Moreover, the hydroxytyrosol hexoside could be released by enzymatic activities and be a source of hydroxytyrosol with high antioxidant power, which is very interesting for future studies. On the other hand, in the solid form, no increase was observed in any compound other than oleuropein, suggesting that the olive leaf powder format protects the antioxidant potential. 

As happened to the olive leaf powder when it was under unfavourable conditions, the aqueous extracts at all temperatures also adjusted to a first-order degradation kinetics (Table 2), showing a lower t_1/2_ than the solid form at RT_s_. 

The temperature of 80 °C showed the shortest t_1/2_ of just over three days, while the one with the longest t_1/2_ was the extract stored at RT_l_ for around 13 days. Although there were no significant differences (*p* > 0.05) between storage at temperatures of 60, 40, RT_l_ and 7 °C, the extracts’ storage at RT_l_ (t_1/2_ of 13 days) was selected as the most suitable due to its easy logistics.

**Figure 5:**
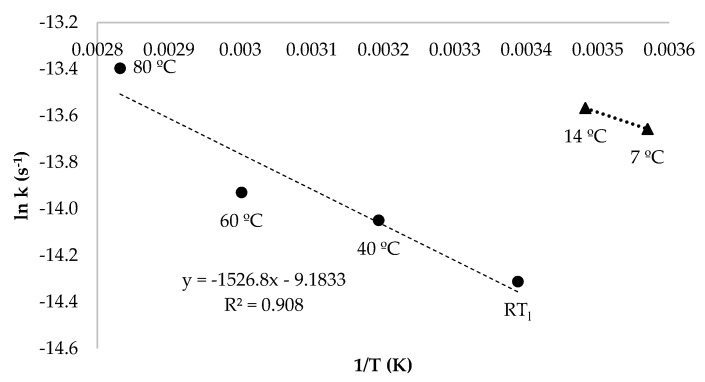
Linear dependence of natural logarithm of the rate constant (ln k, s^−1^) with respect to the inverse of the temperature (1/T, K) of oleuropein content from aqueous extracts stored between temperatures of 80 °C to RT_l_ (24 ± 1.4 °C) and 14 to 7 °C.

After calculating the values of k at different temperatures, the activation energy (E_a_) for oleuropein degradation was obtained through the Arrhenius Equation [25] (Figure 5). It was observed that, from 80 °C to RT_l_, oleuropein degradation was adjusted to a reaction in which E_a_ was 12.60 kJ/mol. Cold temperatures (from 14 to 7 °C) exhibited a different behaviour, thus indicating that a different degradation reaction took place. The latter behaviour has been observed in other antioxidant compounds [15], as the rate of reaction depends on the temperatures.

Regarding the decrease in pH observed in the liquid extracts under storage at 80 °C from 6.30 to 4.42, it is normal that hydroxytyrosol and elenolic acid are released into the medium when oleuropein is degraded. The latter acid may be largely responsible for the decrease in pH, which is attenuated in the other temperatures; however, such acid has not been detected with this method. 

## 5. Conclusions

The temperature, RH and storage form affected the stability of oleuropein content from olive leaves, while illumination had no effect. The degradation process of oleuropein showed better fits in a first order kinetic model with an E_a_ of 12.60 kJ/mol from 80 °C to RT_l_.

Among the storage conditions studied, that which best prevented oleuropein loss was the storage of olive leaf powder below 57% RH at RT_s_, as the aqueous extracts were more unstable. 

The degradation process of oleuropein in aqueous extracts showed similar trends, with the decrease in oleuropein during the first hours followed by an increase in the first week of a glycosylated oleuropein derivative, hydroxytyrosol hexoxide and especially hydroxytyrosol. In the second week, hydroxytyrosol decreased and the glycosylated oleuropein derivative and hydroxytyrosol hexoxide remained stable. In contrast, the degradation of olive leaf powder stored at 75% RH was as follows: oleuropein decreased, and the compounds that increase in the aqueous extracts were not detected. Therefore, olive leaf powder stored in environmental conditions could constitute an interesting opportunity for the olive sector through the re-evaluation of olive leaves transforming them from waste to by-product.

## Figures and Tables

**Table 1:** Rate constants (k) and half-life periods (t_1/2_) of oleuropein content loss in olive leaf powder stored at 75% RH at room temperature (RT_s_) and 40 °C.

T (°C)	k (Days^−1^)	t_1/2_ (Days)
RT_s_^1^	0.023 ± 0.00a	30.137
40 °C	1.282 ± 0.00b	0.541

^1^ Room temperature (22.8 ± 1.9 °C). For each k value, different small letters indicate significant differences among temperatures according to the Tukey test (α < 0.05). The mean values (*n* = 3) are shown with their standard deviation.

**Table 2:** Rate constants (k) and half-life periods (t_1/2_) of oleuropein content loss in aqueous extracts from olive leaves at different temperatures (T).

T (°C)	k (Days^−1^)	t_1/2_ (Days)
80	0.202 ± 0.00a	3.431
60	0.077 ± 0.00b	9.002
40	0.068 ± 0.00b	10.193
RT _l_^1^	0.053 ± 0.00b	13.078
14	0.111 ± 0.00c	6.245
7	0.075 ± 0.00b	9.242

^1^ Room temperature (24 ± 1.4 °C). For each k value, different small letters indicate significant differences among temperatures according to the Tukey test (α < 0.05). The mean values (*n* = 3) are shown with their standard deviation.

## Data Availability

Data is contained within the article.
